# The Role of Ultrasound Guided Sampling Procedures in the Diagnosis of Pelvic Masses: A Narrative Review of the Literature

**DOI:** 10.3390/diagnostics11122204

**Published:** 2021-11-26

**Authors:** Francesca Arezzo, Vera Loizzi, Daniele La Forgia, Adam Abdulwakil Kawosha, Erica Silvestris, Viviana Cataldo, Claudio Lombardi, Gerardo Cazzato, Giuseppe Ingravallo, Leonardo Resta, Gennaro Cormio

**Affiliations:** 1 Obstetrics and Gynecology Unit, Department of Biomedical Sciences and Human Oncology, University of Bari “Aldo Moro”, Piazza Giulio Cesare 11, 70124 Bari, Italy; viviana.cataldo7@gmail.com (V.C.); dr.claudiolombardi@gmail.com (C.L.); gennaro.cormio@uniba.it (G.C.); 2 Obstetrics and Gynecology Unit, Interdisciplinar Department of Medicine, University of Bari “Aldo Moro”, Piazza Giulio Cesare 11, 70124 Bari, Italy; vera.loizzi@uniba.it; 3 SSD Radiodiagnostica Senologica, IRCCS Istituto Tumori Giovanni Paolo II”, Via Orazio Flacco 65, 70124 Bari, Italy; d.laforgia@oncologico.bari.it; 4 Department of General Medicine, Universitatea Medicina si Farmacie Grigore T Popa, Strada Universitatii 16, 700115 Iasi, Romania; adam.akawosha@gmail.com; 5 Gynecologic Oncology Unit, IRCCS Istituto Tumori “Giovanni Paolo II”, Via Orazio Flacco 65, 70124 Bari, Italy; ericasilvestris@gmail.com; 6 Department of Emergency and Organ Transplantation, Pathology Section, University of Bari “Aldo Moro”, Piazza Giulio Cesare 11, 70124 Bari, Italy; gerycazzato@hotmail.it (G.C.); giuseppe.ingravallo@uniba.it (G.I.); leonardo.resta@uniba.it (L.R.)

**Keywords:** ultrasound-guided sampling procedures, fine-needle aspiration cytology, fine-needle aspiration biopsy, tru-cut biopsy, pelvic masses

## Abstract

Ultrasound-guided sampling methods are usually minimally invasive techniques applied to obtain cytological specimens or tissue samples, mainly used for the diagnosis of different types of tumors. The main benefits of ultrasound guidance is its availability. It offers high flexibility in the choice of sampling approach (transabdominal, transvaginal, and transrectal) and short duration of procedure. Ultrasound guided sampling of pelvic masses represents the diagnostic method of choice in selected patients. We carried out a narrative review of literatures regarding the ultrasound-guided methods of cytological and histological evaluation of pelvic masses as well as the positive and negative predictors for the achievement of an adequate sample.

## 1. Introduction

Ultrasound-guided sampling methods are usually minimally invasive techniques applied to obtain cytological specimens or tissue samples, mainly used for the diagnosis of different types of tumors, such as breast [1] and prostate cancers [2]. 

Currently, the available minimally invasive technique for carrying-out cytological examination is fine-needle aspiration cytology (FNAC), and the techniques for obtaining tissue samples for histological examination includes the fine-needle aspiration biopsy (FNAB) and tru-cut biopsy [3].

Other procedures that can be used to guide sampling procedures include computer tomography (CT), magnetic resonance imaging (MRI), and the digitally directed approach [4]. CT-guided percutaneous biopsy is regarded as an efficient and safe procedure in the evaluation of retroperitoneal and abdominal masses, but it has some limitations when the masses are deep within the pelvis due to limited transabdominal accessibility or limited imaging in the osseous pelvic space. MRI is rarely used, regardless of its imaging benefits, because it requires special non-magnetic equipment and experience [5]. The main benefits of ultrasound guidance are its availability. It offers high flexibility in the choice of sampling approach (transabdominal, transvaginal, and transrectal) and short duration of procedure [6]. The lack of radiation is also a benefit when compared with CT guidance. Ultrasound guided tru-cut biopsy also allows precise real-time tissue collection with complete control of the needle tip during the entire procedure. When combined with color doppler imaging, it also facilitates the choice of the part of the tumor that is most suitable for biopsy. An accurate guidance of the needle tip during the entire procedure also reduces complication risks [7].

## 2. Methods

In September 2021, we searched MEDLINE and Scopus for randomized controlled trials; narrative and systematic reviews; meta-analyses; observational studies, either longitudinal or historical; and case series published in English in the last 25 years using keywords ultrasound-guided sampling procedures, fine-needle aspiration cytology, fine-needle aspiration biopsy, tru-cut biopsy, and pelvic masses. For this narrative review, abstracts from 127 manuscripts found in the literature were assessed by two independent authors; of these, 42 (six fine-needle aspiration cytology, 11 fine-needle aspiration biopsy and tru-cut biopsy, 25 ultrasound evaluation of pelvic masses) were included, based on the impact of the latter studies on current patient management.

## 3. Procedures

**Figure 1:**
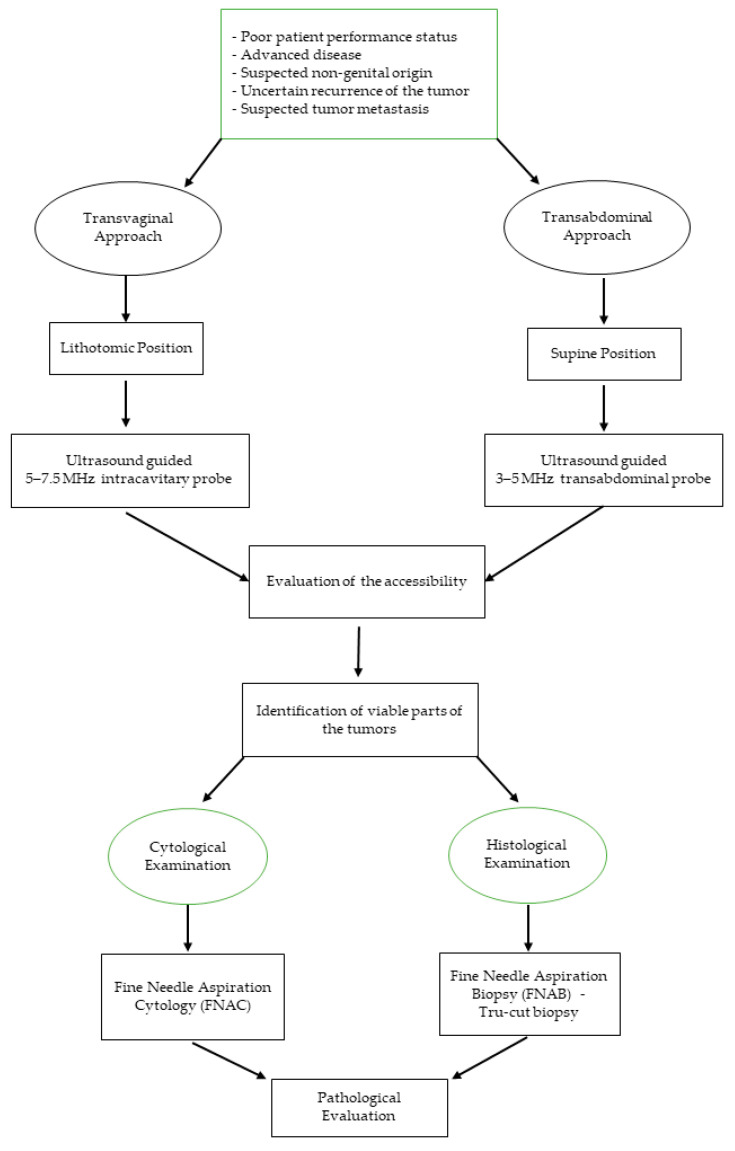
Flowchart on how to utilize ultrasound-guided sampling of pelvic masses.

Normally, the first step in performing an ultrasound-guided sampling is to evaluate the accessibility of the pelvic tumor and then the feasibility of the procedure that could be carried-out through different approaches (transvaginally, transrectally, and/or transabdominally) (Figure 1) [7].

### 3.1. Transvaginal

This procedure is performed in the lithotomy position guided by ultrasound with a 5–7.5 MHz intracavitary transducer with the needle directed to the lesion using a special needle guide attached to an endovaginal ultrasound probe.

The mass location in relation to the vaginal cuff is important in evaluating accessibility via a transvaginal approach. To ensure a good exploration, the mass should be within some centimeters off the vaginal cuff in any single plane of view, and there should be no interposing structures, such as the bowel and bladder [8].

Attention should be paid to identifying viable parts of the tumors detected by blood perfusion on the Doppler imaging, and the tissue samples should be obtained using the single use disposable automatic bioptic gun. The cut penetration should be controlled by setting the stopper to a depth of 10–20 mm.

### 3.2. Transabdominal

This procedure is performed guided by ultrasound using a 3–5 MHz transabdominal probe.

Depending on the gynecological pathology, a higher accuracy of transvaginal biopsies is likely attributable to the proximity of the lesion to the probe and ensures a better capacity for guiding the probe more precisely into the vital parts of the tumor [9].

One to three tissue cores of 10–20 mm length and 1.6–2 mm width should be collected and submitted for histologic analysis, after which they would be fixed and stained appropriately. Samples would be examined for the presence of tumors and classified based on suitability for immunohistochemical staining and tumor characteristics such as the origin, subtype, and grade [10].

At the conclusion of the procedure, it is imperative to obtain post-biopsy images with grayscale and color doppler imaging to assess for post biopsy hemorrhage or other likely complications.

Major or minor complications could arise. The major ones are defined as abnormal postprocedural conditions, such as bleeding, infection, organ injury, and persistent pain that may require further workup or treatment. Minor complications are defined as abnormal findings that resolve spontaneously without sequelae [11], which included procedure-related pain or bleeding that generally do not require medications or treatments.

## 4. Cytological Examination

Fine needle aspiration cytology (FNAC) of adnexal cysts with ultrasound guide is a diagnostic and therapeutic procedure that enables cytological examination and immediate additional treatment, if needed.

When combined with clinical and ultrasound criteria, cytological examination may be a useful additional method used in the assessment of adnexal cysts, despite the fact that the examination has low sensitivity of 44%, specificity (67%), and positive and negative prognostic values of 40% and 71%, respectively.

There is also the risk of malignant cells spreading in an unrecognized way. Therefore, this method is yet to be widely accepted as a diagnostic and therapeutic procedure in pathologic adnexal cases. As a therapeutic procedure, Zanetta et al. reported no significant difference in the recurrence rate of aspirated simple cysts over expectant management (46% and 45%, respectively) [8].

This procedure is not suitable for all cysts that are presumed to be benign. For example, dermoid cyst aspiration is not advised because of the risk of chemical peritoneal granulomatosis and adhesion formation.

The first study about FNAC under ultrasound control of ovarian cysts was published in 1990 (Table 1). Khaw et al. reported about 22 patients with premenopausal unilocular ovarian cysts. No complications were reported. It was the first study that concluded that FNAC is a simple, safe, and useful procedure, and the authors also stated that the surgery can be reserved for cysts with ultrasound suspicion of malignancy, cysts that recur after aspiration, or cystic masses in post-menopause [9].

In 1997, Brunner et al. analyzed FNAC as a simple outpatient strategy for immediate pain relief as well as effective treatment of 26 sterilized patients with ovarian cysts. Similarly, in this case, no complication was observed, and neither was there evidence of malignant cells on cytological examination. Only in a single case, dyskaryotic cells were diagnosed but, after histological examination of the excision of the ovarian cyst was performed, it revealed a benign tumor. In nine patients, the ovarian cysts recurred, and in two patients, surgery were performed [10].

Petrovič et al. in 2001 reported 72 cases of transvaginal ultrasound guided aspiration of adnexal cystic mass. Based on cyst type, 62 were unilocular and 10 multilocular. Fifty-five of these were premenopausal, and 17 were postmenopausal. Disease recurrence was observed in 32 cases (44%) and was more common with larger cysts, while patient’s age did not influence the recurrence rate.

There were complications in one case (1.4%). After aspiration of an endometriotic cyst, an inflammation developed and was treated with antibiotics and aspiration of pus content [11].

Díaz de la Noval et al. performed a study evaluating the effectiveness of ultrasound-guided aspiration for the management of 156 low-risk adnexal cysts. Post treatment follow-up protocol included transvaginal US at 3 and 12 months. The study elicited a complication rate of the procedure of 2.6% (n = 4), with three cases of a major complication attributed to a pelvic abscess and one case of a minor complication of self-limited vaginal spotting. In the study, more than one-third of patients had high-risk comorbidity, although 69.2% (n = 108) avoided surgery, but the overall success rate of US-guided aspiration (resolution rate of the cys) was quite low: only 36.5% [12].

Another transvaginal US-guided aspiration of ovarian cysts was reported by Kostrzewa et al. in 2019. Eighty-four women with simple cysts were analyzed according to the International Ovarian Tumor Analysis (IOTA). They had 100% compatibility with ultrasound diagnosis and cytological examination of aspirated fluid, although, in the study, the cumulative rate of cyst recurrence was high (17/84, 20.02%). The higher percentage was in the premenopausal group, 27% (10/38) vs. 15.2% (7/46) in the postmenopausal group, but the difference was not statistically significant (hazard ratio (HR) = 1.89, 95% confidence interval (95% CI) = 0.72–4.97; *p* = 0.19) [13].

Therefore, it could be ascertained that ultrasound-guided aspiration of adnexal cysts is a diagnostic and therapeutic procedure that enables a cytologic examination. As a treatment option, ultrasound-guided aspiration could be useful in selected cases, such as elderly patients or patients at high risk, with a low rate of resolution, with even 63.5% of recurrence reported by Díaz de la Noval et al. [14,15].

Its usefulness could be applied as a diagnostic approach in certain cases in which the women are high risk for surgery, and it is of interest to avoid it; surgery can be reserved for when it is desirable to obtain a therapeutic result.

## 5. Histological Examination

Fine needle aspiration biopsy (FNAB) is a simple less-invasive biopsy technique, first described by Guthrie in 1921. It is feasible with both 16–18 G needles for the transvaginal approach and 14–16 G needles for the transabdominal procedure [7]. For many years, this has been the standard method for histological verification of tumors in various locations. This method has some limitations, however; the main limitations are the small sample size and the limited amount and integrity of sample tissue collected, often with disrupted tissue architecture, resulting in a high rate of inadequate samples for histological assessment [16].

Moreover, low sample quality may prevent proper immunohistochemistry staining, which could play a crucial role in the differential diagnosis, especially when it involves ovarian or colon cancer [17].

On the other hand, tru-cut biopsy provides a sample with preserved tissue architecture that allows comprehensive histological evaluation as well as permitting the collection of larger tissue samples, thus permitting other vital histological examinations, such as immunohistochemistry [18]. Obviously, this technique would require a larger-sized cutting needle in comparison to FNAB.

Several papers have compared FNAB and tru-cut biopsy. The largest study included 1300 consecutive CT-guided biopsies from chest, abdomen, retroperitoneum, and head/neck regions that evaluated the adequacy and specific diagnosis rates of FNAB (22 G needle) and tru-cut biopsy (16–18 G needle) [19]. Adequate samples were obtained in 72–92% of cases using FNAB and 93–100% using tru-cut biopsy; the specific diagnosis rates were 54–67% and 82–100%, respectively.

In gynecology, in comparison to other diagnostic techniques, this procedure could be a valid alternative method to laparoscopy or laparotomy to obtain histological samples, especially in patients where there are doubts concerning the benefit of a primary surgical intervention (poor patient performance status due to co-morbidities or advanced disease) or the nature of the lesion (uncertain recurrence of the tumor, suspected tumor metastasis, or cases of advanced tumors of probably non-genital origin). 

As reported in Table 2, the first study about FNAB in gynecology was reported in 1991 by Volpi et al. They performed 18 ultrasound-guided biopsies on pelvic masses using 23 cm long 16 or 18 G biopsy needles. One false-negative histological evaluation and one inadequate sample was reported, and no complications were observed [20]. Hence, from the preliminary data, the authors supported that this was a promising technique that is easy to perform and well-accepted by the patients.

In 1997, the accuracy of FNAC and core biopsy in the diagnosis of gynecologic lesions was compared by Malmström, making it the first report about the use of core biopsy in gynecology. A total of 85 patients with persistent, recurrent, or metastatic disease were analyzed using an automatic biopsy instrument (Biopty) simultaneously performing FNAC and core biopsy. Three hundred and thirty-nine FNAC and 141 biopsies using the biopsy core instrument were obtained. A correct diagnosis was made with FNAC in 67/85 (79%) and with core biopsy in 62/85 (73%) of the cases (*p* = 0.08). Insufficient material for evaluation was recorded for FNAC in 12/85 (14%) compared to 10/85 (12%) for the core biopsy (*p* = 0.29). False-negative diagnoses occurred in 5% of the cases with FNAC compared to 15% with core biopsy (NS). The sensitivity of FNAC was 92% and that of core biopsy 73% (*p* = 0.01) and the specificities 92% and 100% (NS), respectively. The predictive values of positive results for the two methods were 96% and 100%, respectively. The complication rate was negligible. In conclusion, FNAC in combination with core biopsy in gynecologic lesions is a simple and safe operation using needle guides. As reported in Table 3, in comparison with FNAC the sensitivity for core biopsy is low but the specificity is high. There were no significant differences in accuracy between the two methods [16].

In 2008, Fiscerova et al. published the first study evaluating the accuracy and safety of ultrasound-guided transvaginal or transabdominal tru-cut biopsy in 86 cases of advanced primary or recurrent pelvic and/or abdominal tumors. Biopsy indications were primarily inoperable pelvic tumor, poor performance status, and recurrent disease requiring histologic verification. The samples collected were from pelvic tumor in 47 cases (54.6%), omental cake in 12 cases (14%), and parietal or visceral carcinomatosis in 27 cases (31.4%). On average, two samples were taken per case (range 1–3). The time taken to obtain two samples of tissue ranged between 10 and 15 min in each patient. Six were inadequate for histologic evaluation. The only complication encountered was bleeding from a tumor in a patient with mild thrombocytopenia, requiring laparotomy. The procedure reached high diagnostic accuracy of 97.7% (95% CI 91.85–99.72%) [21].

Zikan et al. in 2010 analyzed 190 patients with advanced abdominal and pelvic tumors who would not benefit from a primary oncogynecological surgery, patients with history of non-gynecological tumor and who were suspected of having a secondary ovarian or peritoneal tumor. Statistical analysis reported that the single dependent variable was the adequacy of the histological sample from the tru-cut biopsy, while the other variables (age, BMI, CA 125 level, ascites, histology, indication, biopsy site, and biopsy approach) represented the independent factors. Age and BMI did not influence the adequacy of the biopsy sample. Ascites, elevated CA 125, primary suboptimal operable tumor, serous epithelial ovarian cancer histology, carcinomatosis, and vaginal approach were significant positive predictors for the achievement of an adequate sample, while recurrence as an indication, non-serous and non-ovarian histotypes, and a transabdominal approach were negative predictors. Ascites and elevated CA 125 levels were positive predictors for the achievement of an adequate sample for the large tumor load in these cases. Carcinomatosis, as a biopsy site that is often easily accessible transvaginally in the pouch of Douglas and transabdominally by a peritoneal approach through the anterior abdominal wall, also correlated positively with adequacy. The serous histotype of ovarian cancer was a strong positive predictor of adequacy because this histotype is often present as solid nodules of carcinomatosis and a large tumor load in advanced stages. Nonovarian tumors were, on the other hand, a strong negative predictor. This finding cannot be easily explained, whereas inoperable/sub-optimally operable tumors were a favorable indication with a strong positive correlation with adequacy. This finding is in concordance with the abovementioned explanation. A large tumor mass as a biopsy site is usually accessible in these patients, while in patients with recurrent tumor or with tumor of atypical morphology, only small, necrotic, or cystic lesions are often present.

Obesity is also considered a factor impeding the accuracy of ultrasound, thus also potentially affecting the performance of tru-cut biopsy (especially by the abdominal approach).

Two cases of bleeding after tru-cut biopsy (1%) were reported. In the first case, bleeding from the biopsy site on the surface of a Krukenberg tumor caused hemoperitoneum and needed adnexectomy. This patient had a bone marrow infiltration caused by an advanced disseminated tumor that caused thrombocytopenia. We currently consider thrombocytopenia to be a significant contraindication for tru-cut biopsy. In the second patient, ultrasound examination revealed bleeding into the ascitic fluid from the site of the biopsy of pelvic carcinomatosis. A laparoscopy was performed immediately, but, in the meantime, the bleeding stopped spontaneously [22].

Epstein et al. compared subjective ultrasound assessment and the ADNEX (Assessment of Different NEoplasias in the adneXa) model with ultrasound-guided tru-cut biopsy to differentiate disseminated primary ovarian cancer from metastatic non-ovarian cancer. They undertook a prospective study including 143 women with disseminated malignancy of unknown primary origin, with a pelvic tumor/carcinosis. The ultrasound examiner assessed tumor morphology, spread in the pelvis and abdomen, and predicted tumor origin as primary ovarian or metastatic using both subjective assessment and the ADNEX model. Histology from tru-cut biopsy served as the gold standard for assessment of diagnostic accuracy. Subjective ultrasound evaluation had a sensitivity of 82% (73/89) and a specificity of 70% (26/37) in predicting primary ovarian cancer. The ADNEX model had an area under the receiver–operating characteristics curve of 0.891 (95% CI, 0.794–0.946) (in women with an ovarian lesion, n = 104). Subjective ultrasound assessment and the ADNEX model can both be used to predict whether a pelvic tumor is metastatic and of non-ovarian origin, indicating the need for tru-cut biopsy.

They reported two admissions for inpatient care related to biopsy: one patient had an abdominal wall hematoma after transabdominal percutaneous biopsy, and another patient had a suspected pelvic infection after tru-cut biopsy with a transvaginal approach [23].

Park et al. analyzed 55 women with pelvic masses who underwent US-guided transvaginal core biopsy.

The overall diagnostic accuracy of US-guided transvaginal core biopsy was 93% (51/55). Of the 55 lesions, 46 (84%) were confirmed to be either benign or malignant tumors, and five (9%) were diagnosed as active or chronic inflammatory lesions. Four lesions (7%) were not histopathologically diagnosed after biopsy.

In terms of minor complications, vaginal bleeding occurred in 10 patients (18%), and gross hematuria occurred in two patients (4%). These complications resolved spontaneously in all patients without further workup or treatment. They reported that the reliable and accurate diagnostic performance of the procedure might be explained by the ability to advance the needle parallel to the longitudinal axis of the transvaginal probe using an attached guide similar to that used in transrectal prostate biopsy.

The mean gap between the probe and the targeted lesion was only 1.6 cm. The safety and accuracy of the procedure may be explained by the short distance of the biopsy route, although a significant difference between diagnostic and non-diagnostic biopsies was not found [24].

Eitan et al. in 2017 reported 55 patients with undiagnosed pelvic lesions. When it could be measured (*n* = 48), the median size of the lesions biopsied was 43 mm (range 16–130 mm).

Thirty-three lesions were evaluated by FNAB of the solid structure and 26 by aspiration of fluid for cytology. Pathologic feasibility rate was 88% (52/59). Considering the seven inconclusive results, the procedure had sensitivity of 88% (29/33) and specificity of 88% (23/26). Overall accuracy of TVUS-FNA for this patient cohort was 85%. No patient characteristics were found to distinguish between accurate and inaccurate or inconclusive TVUS-FNA result [15].

Also in 2017, Lin et al. analyzed 200 patients affected by primary inoperable tumors, suspicion of metastases to the ovaries or peritoneum, recurrence, or other solid lesions in the pelvis.

A large number of transvaginal biopsies was carried out and reported in this study.

Adequate samples were obtained in 96% of biopsies without any complications.

With a univariate analysis, they evaluated factors that may affect transvaginal ultrasound-guided FNAB success.

The biopsy site had a significant effect on biopsy adequacy with a significantly lower probability of obtaining satisfactory specimens for histologic verification from the peritoneal cake compared to pelvic tumors (83.9% versus 98.5%) (*p* < 0.05) and from the peritoneal cake compared to vaginal cuff masses (83.9% versus 97.1%), (*p* < 0.01). Adequacy was also affected by tumor size (*p* < 0.05) but not by vascularization, ascites, or tumor type [26].

In 2019, Mascilini et al. analyzed imaging of abdominal or pelvic tumors in 62 patients considered not-ideal candidates for primary gynecological surgery (poor performance status, not suitable for surgery, or patients with multiple or non-resectable metastases) or where the origin and/or nature of the tumor was unclear. Of this, ultrasound characteristics were a solid tumor for 61 (98.4%) and a multilocular-solid for 1 (1.6%) (Table 4).

The ultrasound-guided biopsy was mostly performed through the vaginal cuff (25/62, 40.3%). Other sites of biopsy were the cervix (16/62, 25.8%), vaginal wall (11/62, 17.7%), pelvic mass (7/62, 11.3%), and peri-urethral region (3/62, 4.8%).

An adequate sample for histological analysis was obtained in all cases. No major complications were registered. Histopathological examinations showed 24 (38.7%) benign lesions and 38 (61.3%) malignant tumors. Ten patients eventually underwent surgery. Final histology was not in concordance with the results from transvaginal ultrasound-guided biopsy in 2 out of 10 patients (20%); in particular, benign disease at transvaginal ultrasound-guided biopsy was malignant at final histology (two cases of recurrence of cervical cancer) [27].

Gao et al. in 2019 [28] conducted a retrospective analysis on 40 patients with pelvic masses who underwent transvaginal or transrectal ultrasound-guided aspiration biopsy. No complications were identified. The median lesion size was 5.5 cm (range, 1–15 cm). Thirty-four of the lesions were solid while six were cystic. The median number of biopsy cores obtained from each patient was 4.0 (range, 2–7 cores). The specimens were obtained from pelvic cavity and pelvic floor in 18 cases (45%), the vaginal stump in six cases (15%), the cervix in two cases (5%), and the vaginal fornix in 13 cases (32.5%). All the specimens were adequate for histologic evaluation and diagnosis. The study showed detection in almost all the suspected clinical cases and was consistent with a more than 90% adequacy and accuracy.

In 2021, Verschuere et al. analyzed the safety and efficiency of performing transvaginal ultrasound-guided tru-cut biopsy for pelvic masses. The main indication for tru-cut biopsy was suspected disseminated disease or recurrence of malignant conditions.

They reported 155 patients. No major complications occurred in the study. Procedure-related events were limited to moderate blood loss (<50 mL) without the need for treatment in 4.5%. Biopsies were deemed adequate for histological evaluation in 84.3% in which a single tissue cylinder was available for diagnosis. When at least two cylinders were available, diagnostic adequacy increased to >95%.

They also investigated the influence of obesity on the adequacy of transvaginal tru-cut biopsies, but BMI did not seem to affect adequacy, according to the study of Zikan et al. As opposed to the transabdominal approach, the close proximity of the transvaginal probe to the lesions enables tru-cut biopsies even in extreme obesity.

Comparing final histology, the diagnostic accuracy of the tru-cut biopsies was 97.2%. Therefore, the authors concluded that transvaginal tru-cut biopsy of pelvic masses is a safe procedure to perform with high adequacy, but multiple biopsies need to be taken to optimize the amount of tissue for histological examination [29].

**Table 4:** Ultrasound characteristics of patients who underwent histological examination.

Authors	Type of Tumor		Median Diameter of the Lesion		Site of Biopsy
Mascilini et al. [27]	Solid tumor 61 (98.4%), multilocular-solid 1 (1.6%).		31 (10–132) mm		Vaginal cuff (25/62, 40.3%). Cervix (16/62, 25.8%), Vaginal wall (11/62, 17.7%), Pelvic mass (7/62, 11.3%), Peri-uretral region (3/62, 4.8%)
Verschuere et al. [29]	142 (80.7%) solid 8 (4.5%) unilocular-solid 26 (14.8%) multilocular-solid		NA		Ovary 70 (39.8%) Cervix 10 (5.7%) Vagina 4 (2.3%) Pelvic peritoneum 74 (42.0%) Other sites: 7 (4.0%) pararectal, bladder wall, or parametrium
Lin et al. [26]	NA		4.80 ± 2.16 cm		pelvic cavity masses 134/200 (67.0%), vaginal cuff or the vaginal wall 35/200 (17.5%) peritoneal cake 31/200 (15.5%)
Zikan et al. [22]	NA		NA		Pelvic mass 125 (64.1%) Carcinomatosis 41 (21.0%) Omental cake 12 (6.2%) Lymph node 11 (5.6%) Other 6 (3.1%)
Fischerova et al. [21]	NA		NA		Pelvic mass (54.6%) peritoneal visceral or parietal metastases (31.4%) Omental cake (14%).
Epstein et al. [23]	Primary ovarian Solid 48 (53.9%) Multilocular solid 33 (37.1%) Unilocular solid 1 (1.1%) Only carcinomatosis 7 (7.9%) >10 locules 12 (14.6%)	Metastatic non-ovarian Solid 21 (56.8%) Multilocular solid 13 (35.1%) Unilocular solid 0 (0) Only carcinomatosis 3 (8.1%) >10 locules 8 (23.5%)	Primary ovarian 82 (50–110) mm	Metastatic non-ovarian 80 (57–113) mm	

## 6. Conclusions

Ultrasound-guided sampling of pelvic masses is a useful procedure with a lot of pros and some cons. Ad reported in Table 5. It represents a diagnostic method of choice in selected patients. In patients with severely advanced disease who are not deemed ideal for cytoreduction, ultrasound-guided biopsy represents a real-time, convenient, economical, and quick way of obtaining histological diagnosis, usually not requiring any recovery time as after diagnostic laparoscopy or laparotomy and allowing for an early start of neoadjuvant chemotherapy [30].

The other types of patients eligible for this method are patients with high comorbidity burden and those with suspected relapse or atypical tumor morphology with high probability of nonovarian etiology [31,32,33].

Patients whose result of the tru-cut biopsy was non-gynecological malignancy can be directed to the appropriate specialist assessment before performing a surgery.

Complication rate is negligible; no cases were reported by Volpi et al. [20], Eitan et al. [25], Lin et al. [26]. Mascilini et al. [27], or Gao et al. [28]. Two studies reported only a few complications (Fischerova et al. [21] 1/86 (1.2%) and Zikan et al. [22] 2/190 (1%)). Only one study (Park et al. [24]) reported a major complication rate in 12/55 (22%), all being minor; vaginal bleeding in 10/55 (18%); and gross hematuria in 2/55 (4%).

The general clinical condition of the patient must always be taken into account. One of the two cases of complications reported by Zikan et al. [22] was a hemoperitoneum and revealed bleeding from the biopsy site on the surface of the mass. This patient suffered from thrombocytopenia as a consequence of bone marrow infiltration by an advanced disseminated tumor. It would therefore be appropriate to consider thrombocytopenia as a significant contraindication for tru-cut biopsy.

From the analysis of the studies, we also deduce that the risk of an inadequate sample must be considered (8% for Epstein et al. [23], 6% for Zikan et al. [22], 11.9% for Eitan et al. [25], and 8% for Lin et al. [26]). Failure to obtain an adequate sample may be related to cystic or necrotic tumors, which are more common in patients with recurrence or with tumors of atypical morphology at ultrasound than in patients with advanced inoperable tumors [27].

As reported by Lin et al., also the biopsy site had a significant effect on biopsy adequacy with a significantly lower probability of obtaining satisfactory specimens for histologic verification from the peritoneal cake compared to pelvic tumors or vaginal cuff masses [26].

Another risk may be the false evaluation or biopsy-failed diagnosis: 1% for Lin et al. [26], 1.7% Zikan et al. [22], and 20% for Mascilini et al. [27] (but this high number is probably untrue due to the low number of patients who have undergone surgery). Probably, in these patients, the small size of the tumor or the cystic component of the tumor did not allow us to obtain the correct samples [27].

Additionally, the number of samples is related to the adequacy of the technique: Fischerova et al. reported that, with an average two samples, they found an accuracy of 97.7% [21].

Tru-cut biopsy is a simple procedure, but it needs to be undertaken by an experienced operator who has undergone a specific training. This is also demonstrated by the study of Verschuere et al. [29], who reported an increase of the biopsy adequacy over the years, from 75% in 2014 to 88% in 2018. This could be attributed to the operators’ increasing experience with the procedure.

The need for anesthesia is not reported in most studies; therefore, this procedure can be performed in a suitable clinic space, without anesthesia with minimal discomfort. Verschuere et al. [29], from their experience, suggested attention is to be paid during gentle probe manipulation using a sufficient amount of gel to determine the most minimal discomfort.

Therefore, ultrasound-guided sampling procedures, in the hands of an experienced operator, represent a simple and reliable method of extreme help to the gynecologist for the correct diagnosis of gynecological diseases and more and for the correct clinical management of the patient.

## Figures and Tables

**Table 1:** Characteristics of patients undergoing cytological examination.

Author	Year	Population	Type of Tumor	Postmenopausal	Needle Type	N° of Complications	Recurrence	Inadequate Sample	Anesthesia	Prophilactic Antibiotic	Approach
Khaw et al. [9]	1990	24	Unilocular	0	NA	0	NA	NA	NA	NA	Transvaginal/transabdominal
Brunner et al. [10]	1997	26	Unilocular	0	NA	0	9 (34%)	NA	NA	NA	Transvaginal
Petrovič et al. [11]	2001	72	62 (86.1%) unilocular 10 (13.9%) multilocular	17 (27.4%)	18 G	1 (1.4%)	32 (44%)	1	None	None	Transvaginal
Díaz de la Noval et al. [12]	2019	156	Unilocular	90 (57.7%)	17 G	4 (2.6%)	99 (63.5%)	7 (4.5%)	None	None	Transvaginal
Kostrzewa et al. [13]	2019	84	Unilocular	46/84 (54%)	22 G	0	17/84 (20%)	NA	None	None	Transvaginal

NA: not available.

**Table 2:** Characteristics of patients undergoing histological examination.

Author	Year	Population	Type of Tumor	Postmenopausal	Tecnique	Needle Type	N° of Complications	Inadequate Sample		False Evaluation	Anesthesia	Prophylactic Antibiotic	Approach	Accuracy
Volpi et al. [20]	1991	18	Pelvic masses	NA	FNAB	16 G or 18 G	0	1 (5.5%)		1 (5.5%)	NA	NA	transvaginal	NA
Fischerova et al. [21]	2008	86	Advanced primary or recurrent pelvic and/or abdominal tumors	NA	Tru-cut	Transvaginal 16 G, transabdominal 14 G	1 (1.2%)	6 (7%)		2 (3.3%)	Transvaginal: no anesthesia Transabdominal: local anesthesia	NA	Transvaginal 46 (53.5%) Transabdominal 40 (46.5%)	97.7% (95% CI 91.85–99.72%)
Zikan et al. [22]	2010	190	Disseminated malignancy of unknown primary origin	NA	Tru-cut	18 G	2 (1%)	12 (6%)		2/118 (1.7%) patients who underwent subsequent surgery	None	none	Transvaginal transabdominal	98.3%
Epstein et al. [23]	2016	143	Disseminated malignancy of unknown primary origin	NA	Tru-cut	Transvaginal 18 G, Transabdominal 16 G	2 (1.4%)	5 (3.5%)		NA	None	none	Transvaginal, transrectal, transabdominal	NA
Park et al. [24]	2016	55	Pelvic lesions	NA	Tru-cut	18 G	12 (22%)	4 (7%)		NA	Local anesthesia with 1% lidocaine	none	Transvaginal	93%
Eitan et al. [25]	2017	59	Pelvic lesion	NA	33 FNAB 26 FNAC	17 G	0	7 (11.9%)		NA	None	none	transvaginal	85%
Lin et al. [26]	2017	200	Disseminated malignancy of unknown primary origin	NA	FNAB	18 G	0	8 (4%)		2 (1%)	None	none	Transvaginal	NA
Mascilini et al. [27]	2019	62	Disseminated malignancy of unknown primary origin	50 (80.6%)	FNAB	18 G	0	0		2/10 (20%) who underwent surgery	none	none	Transvaginal	NA
Gao et al. [28]	2019	40	Pelvic masses	NA	Tru-cut	18 G	0	0		0	None	none	Transvaginal/transrectal	90%
Verschuere et al. [29]	2021	155	Disseminated malignancy of unknown primary origin	NA	FNAB	18 G	Minor 7 (4.5%) Major 0	Single-tissue cylinder 24 (15.7%)	At least two cylinders 8 (<5%)	NA	none	none	Transvaginal	97.2%

FNAB: Fine Needle Aspiration Biopsy; FNAC: Fine Needle Aspiration Cytology.

**Table 3:** Characteristics of patients undergoing FNAC and tru-cut biopsy.

Author	Year	Population	Type of Tumor	N° of Complications	Inadequate Sample		False Evaluation		Anesthesia	Prophylactic Antibiotic	Accuracy	
Malmström [16]	1997	85	Persistent, recurrent, or metastatic disease	0	FNAC 12/85 (14%)	Core biopsy 10/85 (12%)	FNAC 5%	Core biopsy 15%	None	None	FNAC sensitivity 92% Specificity 92% Predictive positive values 96%	Core biopsy Sensitivity 73% Specificity 100% Predictive positive values 100%

FNAC: fine needle aspiration cytology.

**Table 5:** Pros and cons of each technique.

		Pros	Cons
Histological examination	FNAB	- simple - less invasive	- small sample size - limited integrity of sample tissue collected - high rate of inadequate samples
	Tru-cut biopsy	- larger tissue samples - preserved tissue architecture - possibility of immunohistochemistry examinations - more adequate samples - more specific diagnosis rates	- larger-size cutting needle
Cytological examination	FNAC	- also therapeutic	- possibility of recurrence - not suitable for all types of cyst

FNAB: fine needle aspiration biopsy; FNAC: fine needle aspiration cytology.

## Data Availability

Not applicable.
